# Transmembrane protein 117 knockdown protects against angiotensin-II-induced cardiac hypertrophy

**DOI:** 10.1038/s41440-023-01377-w

**Published:** 2023-07-24

**Authors:** Yi Yang, Xinquan Wang, Peng Yan, Dan Wang, Tao Luo, Yaqiong Zhou, Shichao Chen, Qiting Liu, Jixin Hou, Peijian Wang

**Affiliations:** 1 Department of Cardiology, The First Affiliated Hospital, Chengdu Medical College, Chengdu, 610500 Sichuan China; 2 Sichuan Clinical Research Center for Geriatrics, The First Affiliated Hospital, Chengdu Medical College, Chengdu, 610500 Sichuan China; 3 Key Laboratory of Aging and Vascular Homeostasis of Sichuan Higher Education Institutes, Chengdu, 610500 Sichuan China

**Keywords:** Cardiac hypertrophy, TMEM117, Mitochondria, Oxidative stress

## Abstract

Mitochondrial dysfunction plays a critical role in the pathogenesis of pathological cardiac hypertrophy. Transmembrane protein 117 modulate mitochondrial membrane potential that may be involved in the regulation of oxidative stress and mitochondrial function. However, its role in the development of angiotensin II (Ang-II)-induced cardiac hypertrophy is unclear. Cardiac-specific TMEM117-knockout and control mice were subjected to cardiac hypertrophy induced by Ang-II infusion. Small-interfering RNAs against TMEM117 or adenovirus-based plasmids encoding TMEM117 were delivered into left ventricles of mice or incubated with neonatal murine ventricular myocytes (NMVMs) before Ang-II stimulation. We found that TMEM117 was upregulated in hypertrophic hearts and cardiomyocytes and TMEM117 deficiency attenuated Ang-II-induced cardiac hypertrophy in vivo. Consistently, the in vitro data demonstrated that Ang-II-induced cardiomyocyte hypertrophy significantly alleviated by TMEM117 knockdown. Conversely, overexpression of TMEM117 exacerbated cardiac hypertrophy and dysfunction. An Ang II-induced increase in cardiac (cardiomyocyte) oxidative stress was alleviated by cardiac-specific knockout (knockdown) of TMEM117 and was worsened by TMEM117 supplementation (overexpression). In addition, TMEM117 knockout decreased endoplasmic reticulum stress induced by Ang-II, which was reversed by TMEM117 supplementation. Furthermore, TMEM117 deficiency mitigated mitochondrial injury in hypertrophic hearts and cardiomyocyte, which was abolished by TMEM117 supplementation (overexpression). Taken together, these findings suggest that upregulation of TMEM117 contributes to the development of cardiac hypertrophy and the downregulation of TMEM117 may be a new therapeutic strategy for the prevention and treatment of cardiac hypertrophy.

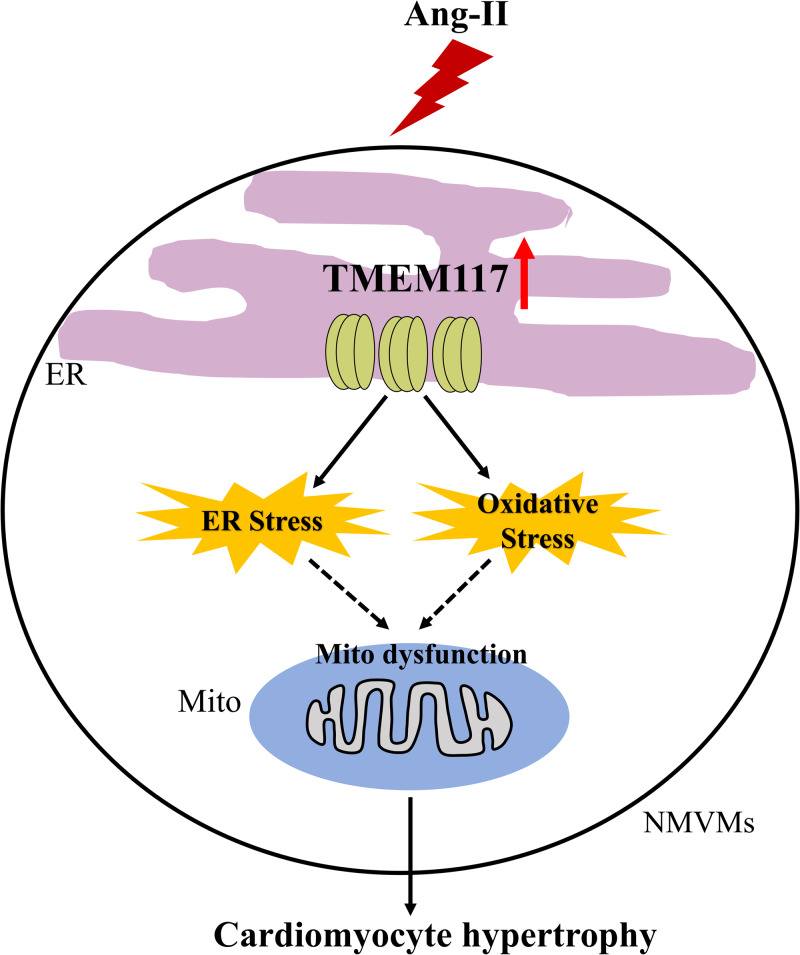

## Introduction

Heart failure (HF), caused by cardiac structural and functional abnormalities, is a global problem, with an estimated 38 million patients with this diagnosis worldwide [1, 2]. Despite the recent development of medical management and evidenced-based therapy, the occurrence of rehospitalization and cardiac events in patients with HF remains high in many countries because most interventions focus on relieving symptoms [3, 4] Cardiac hypertrophy is an early milestone during the clinical course of HF and an important risk factor for subsequent cardiac morbidity and mortality [5, 6]. Cumulative evidence indicates that mitochondrial oxidative stress and dysfunction play an important role in the pathogenesis of cardiac hypertrophy [7, 8]. However, the critical genes that regulate mitochondrial function and that might serve as therapeutic targets for cardiac hypertrophy have yet to be identified.

Transmembrane protein 117 (TMEM117) is a conserved among the chimpanzee, mouse, chicken, frog, and zebrafish, but not in the fruit fly, worm, or yeast. TMEM117 has six transmembrane regions without BCL-2 homology domain, a conserved sequence in pro and anti-apoptotic BCL-2 family proteins, and other known motifs. TMEM117 was first cloned and identified by large-scale cDNA sequencing projects [9, 10]. Previous investigations suggest that TMEM117 expression was downregulated during pancreatic tumorigenesis and transformation and breast cancer cell trans-differentiation to mesenchymal cells [11, 12]. Recent studies reveal that TMEM117 participates in mitochondrial dysfunction, oxidative stress, endoplasmic reticulum stress (ERS), and cell growth impairment [13]. However, the roles of TMEM117 in cardiovascular diseases have not been elucidated. Since mitochondrial dysfunction and oxidative stress are all closely involved with cardiac hypertrophy, we speculate that there might be a chance TMEM117 participates in the process of Ang-II-induced cardiac hypertrophy. The present study investigated the effects of TMEM117 deficiency and upregulation on the development of Ang-II-induced cardiac hypertrophy and underlying mechanism.

## Materials and methods

### Animals

All procedures performed in adherence to the National Institutes of Health Guidelines on the Use of Laboratory Animals (Bethesda, MD, USA) and conformed to the Institutional Animal Care and Use Committee of Chengdu Medical College, the First Affiliated Hospital. Mice were housed in 12/12 h light-dark cycle, a constant room temperature, and fed a standard rodent diet. The C57BL/6J mice (male, 8–10 weeks old) were obtained from the Dashuo Biotech Inc (Chengdu, Sichuan, China). The TMEM117 exon 3 folxed allele was engineered by Cyagen Biotechnology (Guangzhou, China), using CRISPR-mediated homologous recombination. TMEM117^flox/flox^ mice were crossed with Nppa-Cre transgenic mice to obtain cardiomyocyte-specific TMEM117-knockout mice thorough excising specifically exon 3 of the TMEM117 gene in cardiomyocytes. Nppa-Cre transgenic mice were obtained from the Jackson Laboratory (Bar Harbor, USA). TMEM117^flox/flox^ littermates were used as controls.

### Model of angiotensin II infusion

Animals were anesthetized with 2% isoflurane and subjected to pressure overload indued by angiotensin II (Ang-II, Sigma-Aldrich, St. Louis, MO, USA) infusion (1000 ng/kg/min) for 4 weeks. The mini-osmotic pumps (ALZET, model 1004, Cupertino, CA, USA) releasing either Ang-II or 0.9% saline were inserted underneath the mice skin via mid-scapular incision subcutaneously, as our previous studies [14]. Systolic and diastolic blood pressure were measured using a noninvasive tail-cuff apparatus (BP-2010A; Sofron Biotechnology, Beijing, China).

### Neonatal mouse cardiomyocyte culture

Neonatal mouse cardiomyocytes (NMVMs) were prepared form neonatal C57BL/6J mice as previously described [15]. In brief, ventricles were digested with 0.5 mg/mL collagenase (Invitrogen) in a shaking bath at 37 °C. After digestion, cardiomyocytes were collected and cultured in DMEM with 1 g/L glucose, 10% FBS, and 1% penicillin/streptomycin. Then, cells were transfected with small interfering RNA using Lipofectamine RNAiMAX (Life Technologies, Carlsbad, USA) according to the manufacturer’s instructions. Specific small interfering RNAs (siRNAs) against mouse TMEM117 (si*TMEM117*) and scrambled siRNA (si*Control*) were predesigned and purchased from RiboBio (Guangzhou, China). The sequences of siRNA are provided in Supplementary Table 2. To obtain TMEM117-overexpressiong cells, cells were transfected with recombinant adenovirus overexpressing mouse TMEM117 (Ad-TMEM117) or the negative control (Ad-EV) using Lipofectamine 3000 (Life Technologies, Carlsbad, USA) according to the manufacture’s protocol. The coding sequences of TMEM117 were amplified using the primers shown in Supplementary Table 3. After 72 h incubation, the knockdown and overexpression efficiency were detected by western blotting. Subsequently, cells were stimulated with 200 nM Ang-II for 48 h.

### Echocardiography

Transthoracic noninvasive echocardiography was assessed by Vevo 2100 small animal ultrasound system (Toronto, Ontario, Canada). After 28 days of Ang-II infusion, mice were anesthetized with1% isoflurane administered via inhalation though a mask and placed on a heating pad to maintain their body temperature. Then, left ventricle (LV) systolic (LVESd) and diastolic dimensions (LVEDd) were measured in M-mode. LV percent fractional shortening (LVFS) were calculated, as previously described [16].

### Histological analysis

After the mice were euthanized, their heart body (HW), body weight (BW), and tibia length (TL) were determined. From these data, the ratio of HW/BW and HW/TL were calculated. Heart tissues were harvested, fixed in 4% paraformaldehyde, and embedded in paraffin. Tissues were stained with hematoxylin-eosin (H&E) and wheat germ agglutinin (WGA) (green; Sigma-Aldrich) to analyze cardiomyocyte cross-sectional area. Masson staining and Sirius red staining (St. Louis, MO, USA) were used to measure the cardiac interstitial fibrosis and collagen deposition, respectively. The sections were visualized using microscopy, and all images were analyzed using Image-Pro Plus software (Version 6.0).

### Measurement of cell surface area

The cells were fixed by 4% formaldehyde followed by 5 min of permeabilization with 0.2% Triton X-100, blocked with 5% BSA for 60 min and stained with α-actinin (Invitrogen, Carlsbad, CA, USA), followed by staining with a fluorescent secondary antibody. Then, DAPI was used to label the nuclei. Immunofluorescence images were acquired by Olympus laser confocal microscope (FV 1000, Olympus, Tokyo, Japan). Cardiomyocytes surface area was determined from more than 100 cells each group using Image-Pro Plus software (Media Cybernetics, Rockville, MD, USA) by total cell area divided total cell number, as previously described [17].

### Measurement of oxidative stress

Dihydroethidium (DHE) staining was used to evaluate the production of reactive oxygen species (ROS) in NMVMs. Briefly, cells were loaded with DHE (5 μmol/L) (Beyotime, Shanghai, China) in the dark for 30 min and then washed out. Images were taken under an inverted fluorescence microscope with the NIS-Elements 3.2 software (Nikon, Toyoko, Japan), and the mean fluorescence intensity was quantified. In addition, superoxide dismutase (SOD) and glutathione (GSH) and in LV tissue were detected using kits purchased from Beyotime (Beijing, China). Plasma catecholamine (CAT) was measured using the mouse ELISA kit (JiangLai Bio, China).

### Mitochondrial morphology assessment

Heart tissues were fixed with 4% glutaraldehyde in 0.1 mmol/L phosphate-buffered saline (PBS) at 4 °C overnight, followed by another fix with 0.5% potassium ferricyanide and 2% osmium tetroxide in 25 mmol/L cacodylate buffer at 22 °C. Samples were then dehydrated, embedded in resin, and sectioned into 80 nm-thick slices. The sections were observed by JEM-1230 transmission electron microscope (Hitachi H-600IV, Hitachi, Tokyo, Japan). The micrograph images were captured with a digital camera (Olympus, Tokyo, Japan). Mitochondrial mass was analyzed using Image J.

### Assessment of ΔΨm

Mitochondrial membrane potential (ΔΨm) in NMVMs were measured with JC-1 kit (Invitrogen, Carlsbad, CA, USA). Briefly, cells were seeded on culture slides and treated according to experimental protocols. Cells were stained with JC-1 (10 μmol/L) at 37 °C for 30 min and then rinsed three times with PBS. Observations were immediately made using microscope. The ratio of JC-1 aggregate (red) to monomer (green) intensity was calculated.

### Measurement of ATP content

Cellular ATP level was assessed using a commercially available intracellular ATP Assay Kit (Beyotime, Beijing, China). NMVMs were seeded in 6-well plates. After 48 h of Ang-II treatment, cells were harvested for ATP determination according to the manufacturer’s instructions.

### Enzyme assays

Mitochondrial succinate dehydrogenase (SDH) activity was measured in the isolated mitochondria according to the kit’s protocol (Jiancheng Biochemical). Briefly, the mitochondria were incubated with substrate solution and read with a spectrophotometer at 600 nm. The activity of NADPH oxidase was determined by a lucigenin enhanced chemiluminescence assay according to the kit’s protocol (Genmed). Enzyme activity was expressed as RLUs/mg protein.

### Determination of mRNA expression

Total RNAs from myocardium or NMVMs were extracted by TRIzol (Invitrogen, Carlsbad, CA, USA). A total 2 μg of RNA was reverse-transcribed into complementary DNA. The level of mRNAs was quantified by quantitative real-time polymerase chain reaction (qRT-PCR) using SYBRGreen Master Mix (Takara, Dalian, Japan). The target gene expression was normalized to 18 s ribosomal RNA, which served as an internal control for total complementary DNA content. Primer sequences are shown in Supplementary Table 4.

### Measurement of protein expression

Heart samples and myocyte lysates were extracted using lysis buffer. Protein concentration was quantified by bicinchoninic acid (BCA) assay (Bio-Rad, Hercules, CA, USA). Equal amounts of protein (~20 μg) were separated by 10% SDS-PAGE, and then transferred to Polyvinylidene Fluoride (PVDF) membranes (Bio-Rad, Hercules, CA, USA). The membranes were incubated with primary antibodies overnight at 4 °C. More details of antibodies are shown in Supplementary Table 5. The transblotted membranes were washed and incubated with secondary antibodies for 1 h at room temperature. The blots were visualized using chemiluminescence and quantified using Image-Pro Plus 6.0 (Media Cybernetics). Protein expression was normalized to GAPDH.

### Statistical analysis

The data are expressed as means ± SEM. Comparisons between two groups were analyzed using an unpaired Student *t* test. Differences among multiple groups were performed by 1-or 2-way ANOVA using Tukey post hoc test. Differences between groups were analyzed by the Welch *t*-test with multiple comparisons correction (Bonferroni method). *P* < 0.05 was statistically significant. All statistical analysis was performed by GraphPad Prism software version 7.0 (GraphPad Software, La Jolla, CA, USA).

## Results

### TMEM117 was upregulated in Ang-II-induced cardiac hypertrophy

**Fig. 1:**
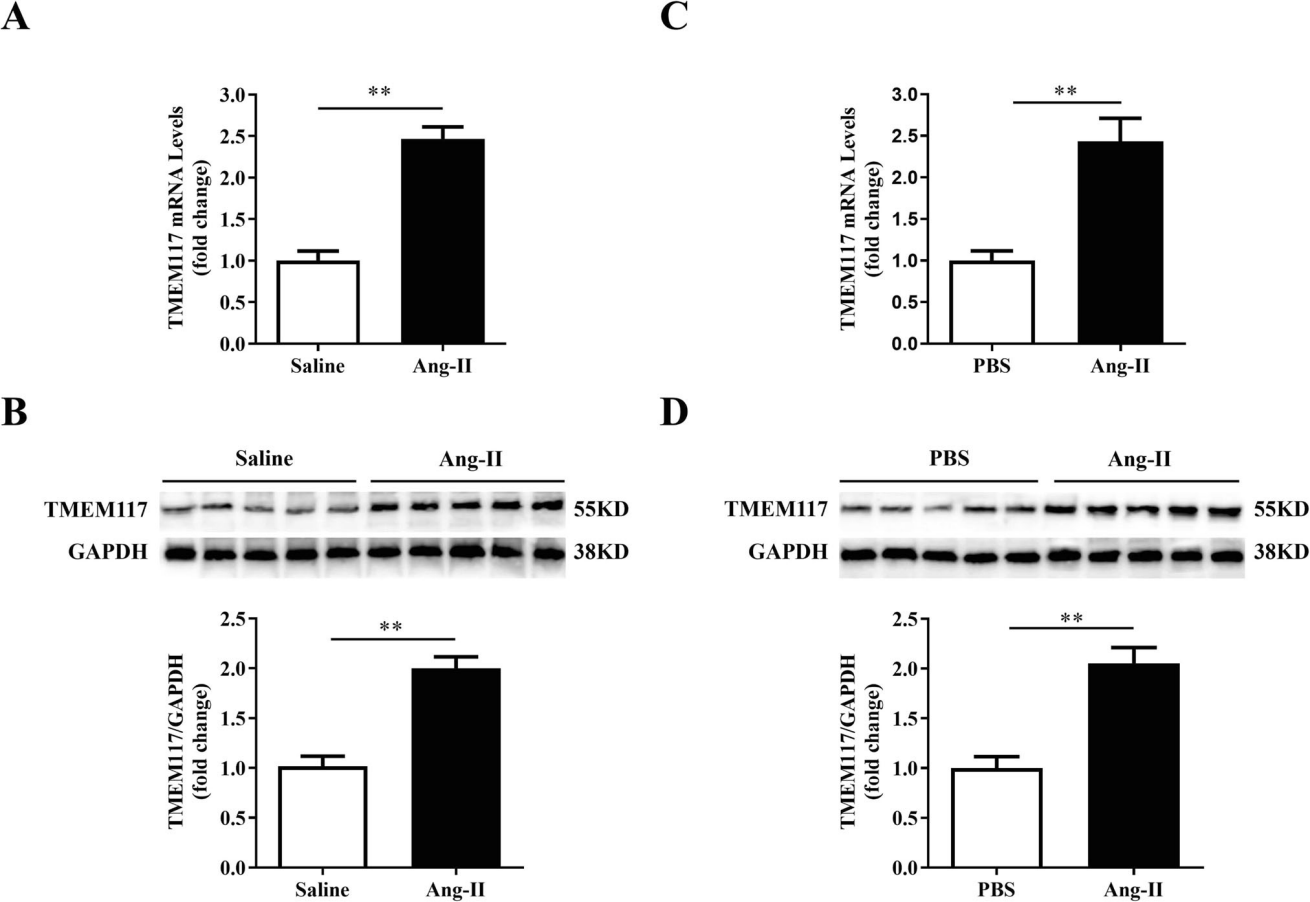
TMEM117 was increased in hypertrophic hearts and isolated NMVMs. **A**,**B** qRT-PCR and western blotting were used to determine the mRNA and protein expression levels of TMEM117 in control and hypertrophic mouse hearts, respectively. **C**,**D** The mRNA and protein levels of TMEM117 in NMVMS with PBS or Ang-II treatment were respectively detected. TMEM117, transmembrane protein 117; GAPDH, glyceraldehyde 3-phosphate dehydrogenase; PBS, phosphate-buffered saline; Ang-II, angiotensin-II. NMVMs, neonatal mouse cardiomyocytes. GAPDH was used as a loading control. Presented values are means ± SEM. *N* = 6–8/group. **P < 0.01 between the 2 indicated groups by two-tailed Student *t* test

To investigate whether TMEM117 is associated cardiac hypertrophy, we first detected the expression of TMEM117 in the hypertrophic hearts and isolated NMVMs. qRT-PCR showed that TMEM117 mRNA levels were remarkably increased in hypertrophic myocardium compared with the Saline group (Fig. 1A). Consistent with changes in mRNA levels, the protein level of TMEM117 was also upregulated in hypertrophic hearts compared to controls (Fig. 1B). We further verified the results in Ang-II-induced cardiomyocyte hypertrophy in vitro. Similarly, both the mRNA and protein expression of TMEM117 were dramatically elevated in NMVMs in response to Ang-II stimulation compared with the PBS group (Fig. 1C,D). Collectively, these findings indicated that TMEM117 might take part in the Ang-II-induced cardiac hypertrophy.

### TMEM117 deficiency ameliorated Ang-II-induced cardiac hypertrophy

**Fig. 2:**
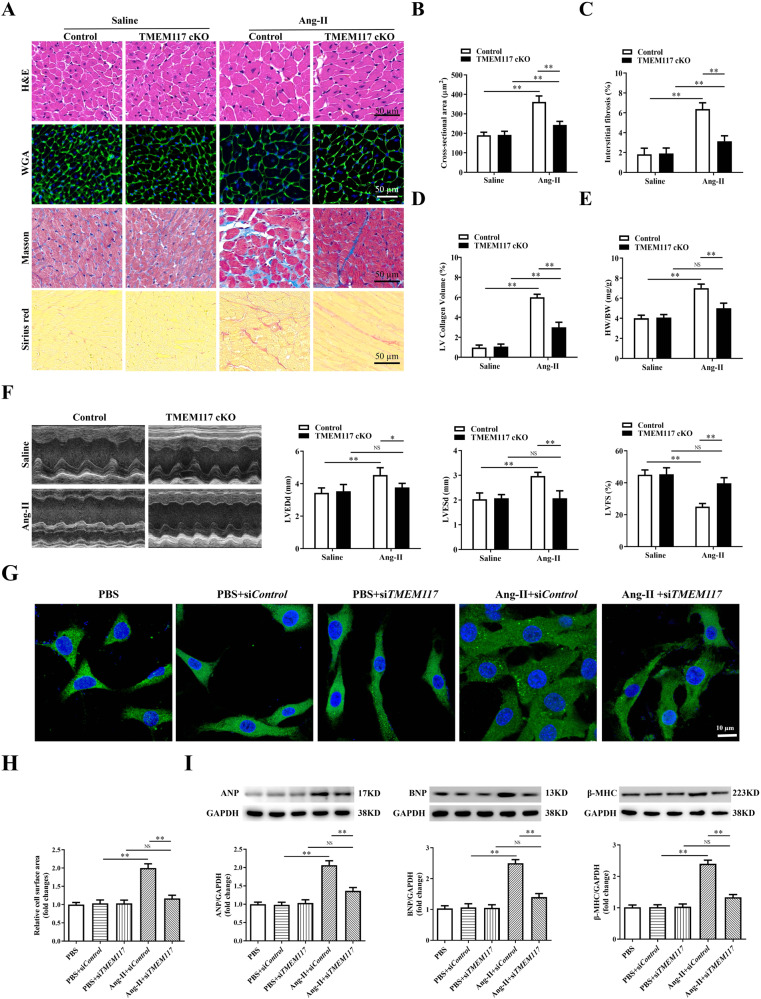
TMEM117 deficiency attenuated Ang-II-induced cardiac hypertrophy. **A** Histological analyses of heart sections stained with H&E, wheat germ agglutinin, Masson and picrosirius red from control mice and TMEM117 cKO mice after saline or Ang-II infusion. **B** The average cross-sectional area of cardiomyocytes was summarized after saline or Ang-II infusion in control and TMEM117 cKO mice. **C**,**D** The interstitial fibrosis and collagen deposition were quantified in the different mice, respectively. **E** HW/BW ratios were detected in the indicated groups. **F** Echocardiographic assessment of LVEDd, LVESd and FS was used to reflect cardiac function in control mice and TMEM117 cKO mice after saline or Ang-II infusion. **G** Representative immunofluorescence images of α-actinin staining in neonatal mice ventricular myocytes infected with si*Control* or si*TMEM117* and treated with or without Ang-II for 48 h. **H** Pooled data from (**G**). **I** Western blotting was used to measure protein levels of ANP, BNP and β-MHC in the indicated groups. ANP atrial natriuretic polypeptide, BNP brain natriuretic polypeptide, β-MHC myosin chain heavy β, Col1 collagen type 1, Col3 collagen type 3, HW/BW heart weight/body weight, LVEDd left ventricular end-diastolic dimension, LVESd left ventricular end-systolic dimension, FS fractional shortening, cKO conditional knockout. All the data represent the means ± SEM. *N* = 6–8/group. ^*^P < 0.05 and ^**^P < 0.01. NS indicates no significance. Statistical significance was determined by 1-way ANOVA or 2-way ANOVA using Tukey post hoc test

To clarify the functional role of TMEM117 in cardiomyocytes during Ang-II-induced cardiac hypertrophy, we generated cardiomyocyte-specific conditional TMEM117-knockout (TMEM117 cKO) mice. We confirmed loss of TMEM117 protein expression in cardiomyocytes of TMEM117 cKO mice (Supplementary Fig. 1A) and did not find any changes in basal physiological parameters in TMEM117 cKO mice and control mice (Supplementary Table 1). Furthermore, TMEM117 cKO and littermate control mice were subjected to Ang-II infusion. Four weeks after Ang-II infusion, the results showed that the systolic blood pressure (SBP) and diastolic blood pressure (DBP) were significantly increased in both mouse models after Ang-II infusion. Blood pressure was indistinguishable between TMEM117M cKO and control mice (Supplementary Fig. 1B,C), suggesting that TMEM117 do not affect Ang-II-induced hypertension. Histological analysis (Fig. 2A) revealed that the cross-sectional area of cardiomyocytes (Fig. 2B), interstitial fibrosis (Fig. 2C), and collagen deposition (Fig. 2D) were obviously larger in TMEM117 cKO mice than of control mice 4 weeks after Ang-II infusion. The heart weight/body weight ratios (Fig. 2E) and heart weight/tibia length ratios (Supplementary Fig. 1D) were significantly decreased in TMEM117 cKO mice compared with control mice in response to hypertrophic stimuli. In addition, TMEM117 deficiency attenuated cardiac function in the hypertrophic hearts, evidenced by decreased left ventricular end-diastolic dimension (LVEDd) and left ventricular end-systolic dimension (LVESd) and increased fractional shortening (FS) (Fig. 2F). Consistently, the expression of hypertrophic genes, including ANP (atrial natriuretic polypeptide), BNP (brain natriuretic peptide), β-MHC (myosin heavy chain β), and fibrotic genes, including Col1 (collagen type 1) and Col3 (collagen type 3) (Supplementary Fig. 1E) were decreased in the hypertrophic hearts of TMEM117 cKO mice.

We next investigated whether TMEM117 directly regulates cardiomyocyte hypertrophy using an in vitro model of cardiomyocyte hypertrophy induced by Ang-II in NMVMs, which isolated from left ventricular in mice hearts. TMEM117 in NMVMs was silenced by siRNA and treated with or without hypertrophic stimulation (Supplementary 1F–H). NMVMs with TMEM117 knockdown had a dampened hypertrophic response to Ang-II treatment based on the restoration of cell surface area (Fig. 2G,H) and decreased expression of protein levels of ANP, BNP and β-MHC (Fig. 2I). There are no differences of ANP, BNP and β-MHC levels between PBS and PBS with si*Control*. Taken together, these observations demonstrated that TMEM117 deficiency ameliorated cardiomyocyte hypertrophy.

### Cardiac TMEM117 overexpression exacerbated Ang-II-induced cardiac hypertrophy

**Fig. 3:**
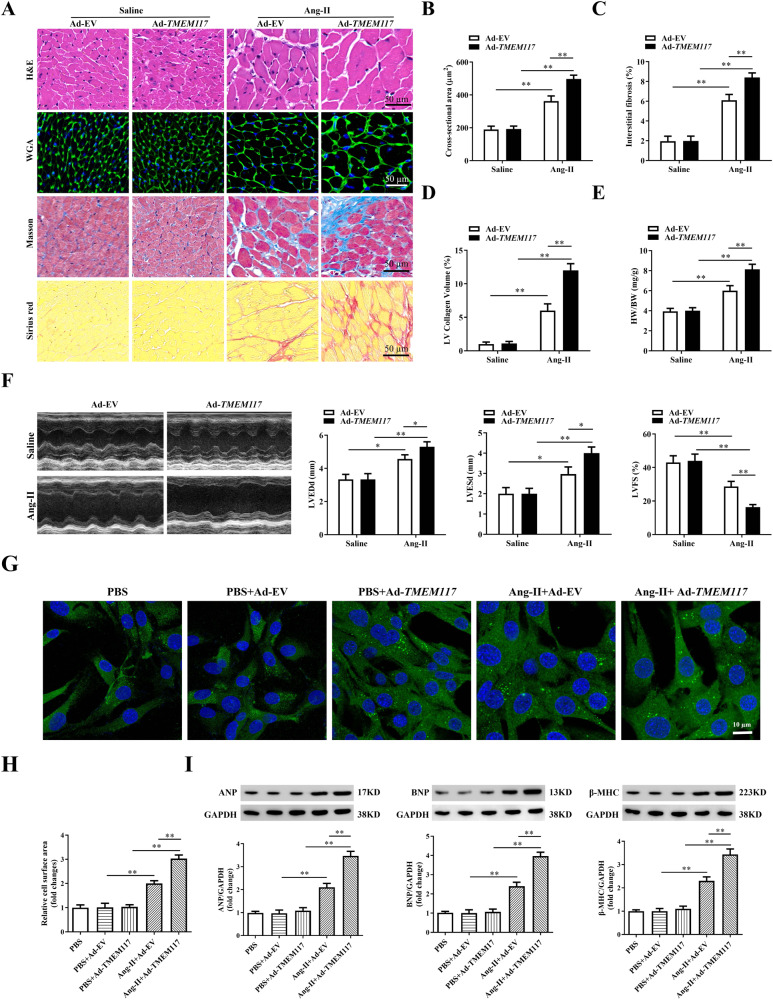
TMEM117 overexpression in the heart deteriorated cardiac hypertrophy. **A** Heart sections were stained with H&E, wheat germ agglutinin, Masson and picrosirius red from control mice injected with Ad-EV or Ad-*TMEM117* subsequently subjected to saline or Ang-II. **B** The average cross-sectional area of cardiomyocytes from the indicated groups was summarized. **C**,**D** The interstitial fibrosis and collagen deposition were quantified. **E** The rations of HW/BW in different mice were determined. **F** Echocardiographic assessment of LVEDd, LVESd and FS in the indicated groups. **G** Representative immunofluorescence images of α-actinin staining in neonatal mice ventricular myocytes infected with Ad-EV or Ad-*TMEM117* and treated with or without Ang-II for 48 h. Scale bars = 10 μm. **H** Pooled data from (**G**). **I** Western blotting was used to measure protein levels of ANP, BNP and β-MHC in the indicated groups. Ad-EV, control adenovirus; Ad-*TMEM117*, recombinant adenovirus encoding TMEM117. All the data represent the means ± SEM. *N* = 6–8/group. ^*^P < 0.05 and ^**^P < 0.01. NS indicates no significance. Statistical significance was determined by 1-way ANOVA or 2-way ANOVA using Tukey post hoc test

To further explore the effects of TMEM117 in cardiomyocytes on cardiac hypertrophy, an adenoviral vector expressing TMEM117 was administered through direct injection into left ventricle in control and hypertrophic mice. Following adenoviral injection, TMEM117 expression in ventricular myocardium was confirmed by using western blotting analysis (Supplementary Fig. 2A,B). Basal physiological parameter was indistinguishable in between TMEM117 overexpression and control mice (Supplementary Table 1). Cardiac TMEM117 overexpression deteriorated Ang-II-induced cardiac hypertrophy, as indicated by enlarged cardiomyocytes area, increased interstitial fibrosis and collagen deposition (Fig. 3A–D), as well as elevated HW/BW (Fig. 3E) and HW/TL ratios (Supplementary Fig. 2C). Furthermore, we observed increased LVEDd and LVESd and decreased FS in TMEM117 overexpression mice with Ang-II infusion compared with control mice, indicating exacerbated cardiac function (Fig. 3F). Meanwhile, the expression of hypertrophic and fibrotic genes was increased in the hypertrophic hearts of TMEM117-overexpressing mice (Supplementary Fig. 2D).

To evaluate the impact of TMEM117 on cardiomyocyte hypertrophy in vitro, TMEM117 was overexpressed in NMVMs with adenovirus (Supplementary Fig. 2E,F). The adenovirus-mediated overexpression of TMEM117 led to a clear increase in cell surface area (Fig. 3G, H). Consistently, Ang-II-induced protein level of the hypertrophic marker genes ANP, BNP and β-MHC were markedly elevated by TMEM117 overexpression compared with controls (Fig. 3I). Taken together, these data indicated that cardiac TMEM117 overexpression aggravated Ang-II-induced cardiac hypertrophy.

### TMEM117 knockout reduced oxidative stress induced by cardiac hypertrophy

**Fig. 4:**
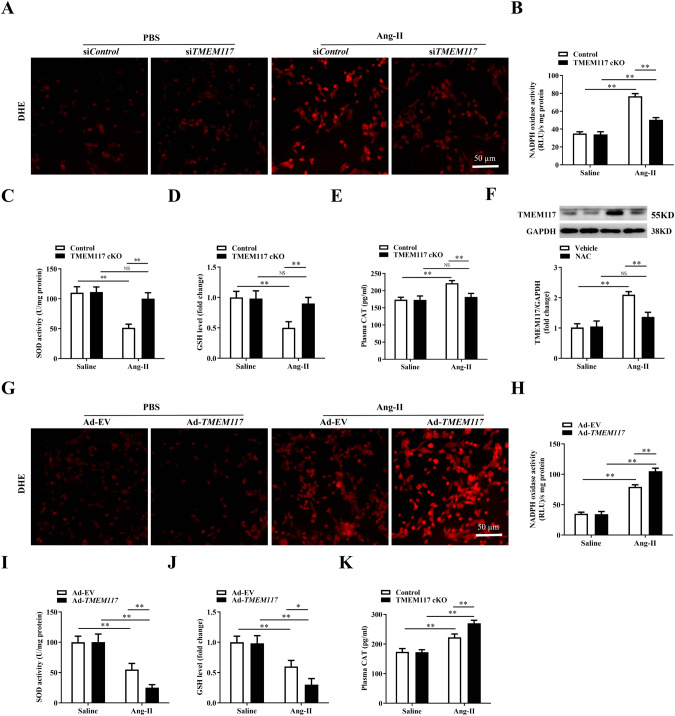
TMEM117 mediated cardiomyocyte hypertrophy by modulating oxidation states. **A** DHE staining was performed in NMVMs infected with si*Control* or si*TMEM117* and treated with or without Ang-II. **B** NADPH oxidase activity was measured in the indicated groups. **C**–**E** Quantitative results of SOD activity and GSH, and plasma CAT levels in control or TMEM117 cKO mice treated with saline or Ang-II. **F** TMEM117 protein level was measured in control and hypertrophic myocardium with or without NAC. **G** Representative DHE staining in neonatal mice ventricular myocytes infected with Ad-EV or Ad-*TMEM117* and treated with or without Ang-II. **H** NADPH oxidase activity was measured in the indicated groups. **I**–**K** Quantitative results of SOD activity and GSH, and CAT levels from control mice injected with Ad-EV or Ad-*TMEM117* subsequently subjected to saline or Ang-II. Presented values are means ± SEM. *N* = 6–8/group. ^*^P < 0.05 and ^**^P < 0.01. NS indicates no significance. Statistical significance was determined by 1-way ANOVA or 2-way ANOVA using Tukey post hoc test

Oxidative stress is a common mechanism underlying the pathological cardiac hypertrophy. We further examined the relationship between TMEM117 and oxidative stress in Ang-II-induced cardiac hypertrophy. DHE staining was used to evaluate in vivo oxidative stress levels. As shown in Fig. 4A, Ang-II induced an overt elevation of ROS generation in NMVMs, which was attenuated by TMEM117 cKO. NADPH oxidase activity was also increased in Ang-II-induced hypertrophic myocardium, which was decreased by TMEM117 deficiency (Fig. 4B). In addition, the results showed that TMEM117 deficiency markedly increased SOD activity (Fig. 4C) and GSH levels (Fig. 4D) in hypertrophic myocardium and decreased plasma CAT levels (Fig. 4E). Furthermore, ROS scavenger N-Acetyl-L-cysteine (NAC) was used in our study. As a result, we found that the increased expression of TMEM117 in hypertrophic myocardium was reversed by NAC treatment (Fig. 4F). To confirm the injury-mitigation roles of oxidative stress and TMEM117 deficiency, we investigated effects of TMEM117 overexpression on oxidative stress in cardiac hypertrophy. As a result, TMEM117 overexpression remarkedly exacerbated oxidative stress, as evidenced by increased ROS generation (Fig. 4G), elevated NADPH oxidase activity (Fig. 4H), decreased SOD activity (Fig. 4I) and GSH levels (Fig. 4J), and increased CAT levels (Fig. 4K). Thus, the in vivo and in vitro data indicated that antioxidative effect of TMEM117 cKO in Ang-II-induced cardiac hypertrophy.

### TMEM117 deficiency attenuated cardiac hypertrophy-induced ERS

**Fig. 5:**
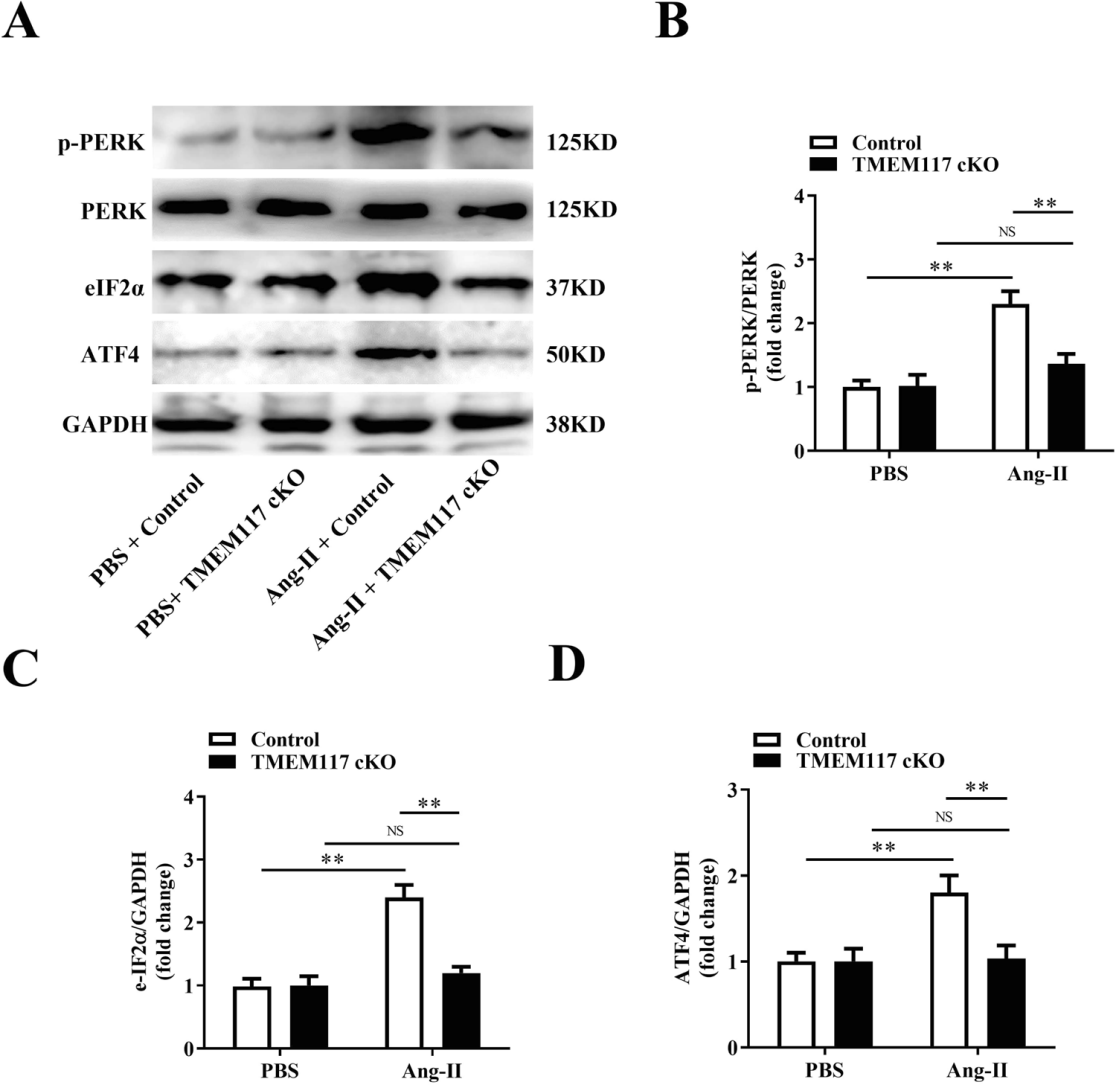
TMEM117 deficiency attenuated cardiac hypertrophy-induced ERS in vivo. **A** NMVMs were isolated from cardiac tissue in Control and TMEM117 cKO mice and cultured in PBS and Ang-II medium. Representative western blot for p-PERK/PERK (**B**), e-IF2α (**C**) and ATF4 (**D**) in NMVMs. PERK, dsRNA-activated protein kinase like ER kinase; p-PERK phosphorylated dsRNA-activated protein kinase like ER kinase, eIF2α eukaryotic initiation factor 2α, ATF4 activating transcriptional factor. All the data represent the means ± SEM. *N* = 6–8/group. ^*^P < 0.05 and ^**^P < 0.01. NS indicates no significance. Statistical significance was determined by 1-way ANOVA or 2-way ANOVA using Tukey post hoc test

Accumulating evidence suggests that protein folding and generation of ROS as a byproduct of protein oxidation in the ER are closely linked events [18]. Other study has shown that TMEM117 is in endoplasmic reticulum and plasma membrane [19]. Therefore, we next focused whether TMEM117 regulates cardiac hypertrophy by modulating ERS-mediated ROS generation. Several markers of ERS were detected in cardiomyocytes from control and TMEM117 cKO mice hearts response to Ang-II stimulation. Western blotting analysis showed that TMEM117 ablation in NMVMs attenuated ERS induced by Ang-II (Fig. 5A), as evidenced by significantly decreased protein levels of phosphorylated dsRNA-activated protein kinase like ER kinase (p-PERK) (Fig. 5B), eukaryotic initiation factor 2α (eIF2α) (Fig. 5C) and activating transcriptional factor (ATF4) (Fig. 5D), indicating that TMEM117 is involved in controlling PERK-mediated ERS pathway in cardiomyocyte hypertrophy.

### TMEM117 deficiency ameliorated cardiac hypertrophy-induced mitochondrial injury

**Fig. 6:**
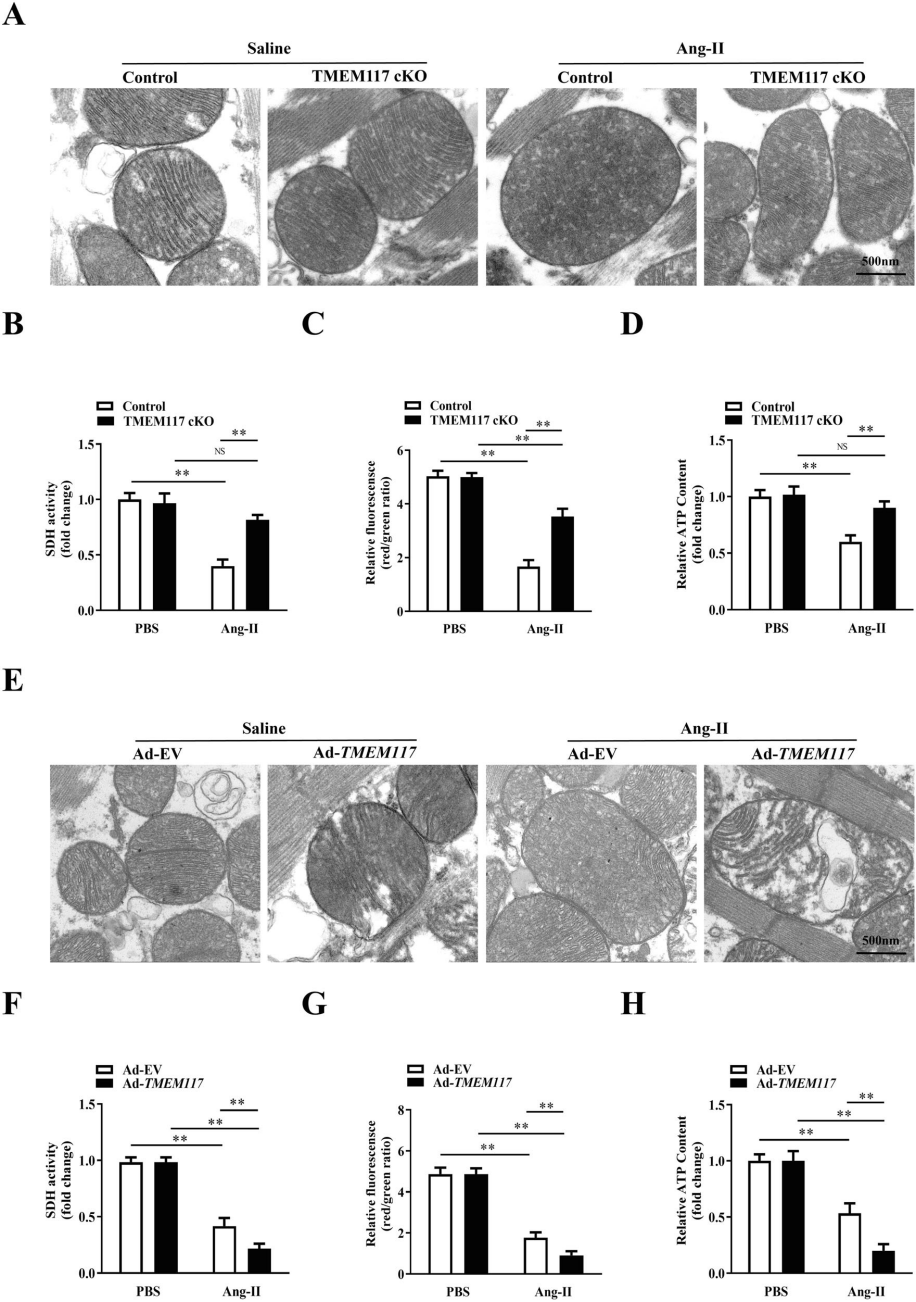
TMEM117 deficiency attenuated mitochondrial disorder induced by cardiac hypertrophy. **A** Transmission electron microscopy was used to observe the mitochondrial morphology in control or TMEM117 cKO mice treated with saline or Ang-II. **B**–**D** Quantitative results of SDH activity, mitochondrial membrane potential and ATP production in NMVMs infected with si*Control* or si*TMEM117* and treated with or without Ang-II. **E** Representative electron microscopy images from control mice injected with Ad-EV or Ad-*TMEM117* subsequently subjected to saline or Ang-II. **F**–**H** Quantitative results of SDH activity, mitochondrial membrane potential and ATP production in NMVMs infected with Ad-EV or Ad-*TMEM117* and treated with or without Ang-II. All the data represent the means ± SEM. *N* = 6–8/group. ^*^P < 0.05 and ^**^P < 0.01. NS indicates no significance. Statistical significance was determined by 1-way ANOVA or 2-way ANOVA using Tukey post hoc test

ROS are primarily generated in mitochondria, and mitochondrial abnormalities induce ROS overproduction. Transmission electron microscopy revealed that TMEM117 cKO attenuated hypertrophy-induced destruction of mitochondrial structure, as evidenced by loss of mitochondrial membranes integrity, unusual vesicle-like structures, completely unstructured cristae, and ambiguous myofilaments (Fig. 6A) in vivo. At the same time, we also found that TMEM117 deficiency alleviated the decreased activity of SDH activity (Fig. 6B), depression of mitochondrial membrane potential (ΔΨm) (Fig. 6C), and suppression of ATP content (Fig. 6D) in NMVMs response to Ang-II. In contrast, TMEM117 overexpression aggravated abnormal mitochondrial morphology (Fig. 6E) and mitochondrial dysfunction (Fig. 6F–H) induced by cardiac hypertrophy. Therefore, these observations suggested that TMEM117 deficiency is crucial for the maintenance of mitochondrial morphology and function, and TMEM117 overexpression exacerbated mitochondrial injury in Ang-II-induced cardiac hypertrophy.

## Discussion

In the present study, we have made several new observations. First, we demonstrated that TMEM117 was upregulated in hypertrophic hearts and cardiomyocytes. Second, loss-of-function and gain-of-function experiments demonstrated that TMEM117 knockdown attenuated, while TMEM117 overexpression aggravated, cardiac hypertrophy induced by Ang-II both in vivo and in vitro. Third, the cardioprotective effects of TMEM117 deficiency in cardiac hypertrophy were associated with suppressed oxidative stress, ERS and improved mitochondrial function, which were abolished by TMEM117 supplementation. Taken together, these findings suggest that the deficiency of TMEM117 protects against cardiac hypertrophy through ameliorating mitochondrial injury and oxidative stress.

Recent studies have found that TMEM117 is a regulator of cell death through apoptosis. TMEM117 is expressed in a variety of normal tissues and organs, and it is mostly involved in the physiological and pathological processes of cancers [11]. Previous studies indicated that TMEM117 was downregulated in tumors and its downregulation was suppressed during the phenotypic change to normal cells in gliomas [20]. Malignant lymphoblastic leukemia has shown low levels of expressed TMEM117 [21]. Other study demonstrated that knockdown of TMEM117 was associated with cell growth impairment and could alter homeostasis toward cell death in HCT116 cells [19]. These research results indicate that TMEM117 plays an important role in the development of cancer. Prior to these findings, little was known regarding the function of TMEM117. Until now, the precise role of TMEM117 in cardiovascular disease is not known. Here, our data showed that TMEM117 was increased in hypertrophic myocardium and NMVMs. Thus, we hypothesized that TMEM117 might serve as the major molecule in the development of myocardial hypertrophy. With genetic method, we found that TMEM117 deficiency attenuated Ang-II-induced cardiac hypertrophy both in vivo and in vitro. In contrast, the overexpression of TMEM117 aggravated cardiac hypertrophy. Specially, blood pressure was indistinguishable between TMEM117 knockdown and control mice, suggesting that TMEM117 do not affect Ang-II-induced hypertension. Previous study show that direct Ang-II effect without hypertension in known to induced myocardial hypertrophy via TGF-β upregulation [22]. We speculated that TMEM117 may mediate direct Ang-II-induced cardiac hypertrophy rather than hypertension-induced hypertrophy. We need more experiments to confirm this hypothesis. Taken together, this demonstrates that deficiency of TMEM117 protects against Ang-II-induced cardiac hypertrophy.

Oxidative stress occurs when ROS are generated that cannot be adequately countered by intrinsic antioxidant systems [23, 24]. Excessive ROS generation triggers cell dysfunction, lipid peroxidation, and DNA mutagenesis and can lead to cell damage or death [25]. ROS has been identified as one of the key contributing factors in the development of cardiac hypertrophy [26, 27]. Cumulative evidence suggests that Ang-II increase mitochondrial ROS levels in cardiomyocytes, and mitochondrial oxidative stress contributes to Ang-II-mediated cardiac hypertrophy [28]. Therefore, we examined the specific role of oxidative stress and mitochondrial function in cardiac hypertrophy. TMEM117 is an endoplasmic reticulum (ER)-associated protein found in different species. Previous studies reported that TMEM117 knockdown led to ΔΨm loss, increased ROS generation, upregulation of ERS sensor C/EBP homologous protein and active caspase-3 expression in HCT116 cells [19]. Contrary to these reports, the present study demonstrated that TMEM117 deficiency attenuated, while upregulation of TMEM117 aggravated, the oxidative stress and ERS of cardiomyocytes in vitro and in vivo. We also found that knockout of TMEM117 protected mitochondrial morphology and function in Ang-II-induced cardiac hypertrophy. Collectively, these results supported the conclusion that reduced TMEM117 alleviated abnormal mitochondrial morphology and function, inhibiting oxidative stress, and ameliorating pathological cardiac hypertrophy.

There are certainly some limitations and problems in this study. The mechanism underlying the upregulation of TMEM117 in cardiac hypertrophy are still unknown. This will call for a lot of follow-up work to make a deeper understanding. We have constructed TMEM117 transgenic mice in our lab and in the future, we certainly will conduct more research and exploration on the role of TMEM117 in the process of cardiac hypertrophy.

In summary, we demonstrate a novel role of TMEM117 in cardiac hypertrophy. The present study provides evidence that upregulation of TMEM117 contributes to the development of cardiac hypertrophy and TMEM117 deficiency attenuates cardiac hypertrophy, likely through ameliorating mitochondrial injury and oxidative stress.

### Supplementary information

Supplementary Materials
