# Supplementary material for: Transmembrane protein 117 knockdown protects against angiotensin-II-induced cardiac hypertrophy
Source: Hypertens Res. 2023 Jul 24;46(10):2326–39. doi: 10.1038/s41440-023-01377-w (PMC10550824; doi:10.1038/s41440-023-01377-w)

**Supplementary Tables**

**Supplementary Table 1 Basal physiological parameters in all kinds of mice**

| **Parameter** | **Control** | **TMEM117 cKO** | | **TMEM117 overexpression** | | |
| --- | --- | --- | --- | --- | --- | --- |
| HR (bpm) | 413±24 | | 418±21 | | 402±27 |  |
| HW/BW (mg/g) | 4.0±0.3 | | 4.2±0.2 | | 4.1±0.3 |  |
| HW/TL (mg/cm) | 60±8.2 | | 63±9.2 | | 60±8.4 |  |
| LVEDd (mm) | 3.5±0.7 | | 3.6±0.4 | | 3.5±0.1 |  |
| LVESd (mm) | 2.1±0.2 | | 2.3±0.4 | | 2.2±0.3 |  |
| FS (%) | 45±2.2 | | 47±3.2 | | 45±1.8 |  |

**Note:** TMEM117 cKO, TMEM117 conditional knockout; HR, heart rate; HW/BW, heart weight to body weight; HW/TL, heart weight to tibia length; LVEDd, left ventricular end-diastolic dimension; LVESd, left ventricular end-systolic dimension; FS, fractional shortening.

**Supplementary Table 2 Sequences of siRNA**

| **Product number** | **Product name** | **Target sequence** |
| --- | --- | --- |
| siG2204180119020056 | si-m-TMEM117_001 | GATATGATGCTTCAAGACA |
| siG2204180119021186 | si-m-TMEM117_002 | GCTACTGGCCATTCTCATA |
| siG2204180119022206 | si-m-TMEM117_003 | GGATCAGCTGGGACAAACT |

**Note:** TMEM117, transmembrane protein 117.

**Supplementary Table 3 Sequences of primers used in gene cloning**

| **Gene symbol** | | **Primers sequences** | | **Product size (bp)** |
| --- | --- | --- | --- | --- |
| TMEM117 | forward primer | | CGCAAATGGGCGGTAGGCGTG  1545 | |
|  | reverse primer | | CATGTGTCGCTGGGAGAGAGGC | |

**Supplementary Table 4 Primer sequences used for PCR**

| **Gene** | | **Forward (5’-3’)** | **Reserve (5’-3’)** |
| --- | --- | --- | --- |
| **TMEM117** | ACGGGAACATGGGAGCTTAT | | GTCAGGGTAGGGCTTGTCTT |
| **ANP** | GTGTACAGTGCGGTGTCCAA | | ACCTCATCTTCTACCGGCAT |
| **BNP** | GAGGTCACTCCTATCCTCTGG | | GCCATTTCCTCCGACTTTTCT |
| **β-MHC** | CCGAGTCCCAGGTCAACAA | | CTTCACGGGCACCCTTGGA |
| **Col1** | ATGGATTCCCGTTCGAGTAC | | ATGGATTCCCGTTCGAGTAC |
| **Col3** | CCCAACCCAGAGATCCCATT | | CCCAACCCAGAGATCCCATT |
| **18sRNA** | CGCGGTTCTATTTTGTTGGTTT | | GCGCCGGTCCAAGAATTT |

**Note:** TMEM117, transmembrane protein 117; ANP, atrial natriuretic peptide; BNP, brain natriuretic peptide; β-MHC, myosin heavy chain; Col1, collagen type 1; Col3, collagen type 3.

**Supplementary Table 5 Primary antibodies used for western blot and immunofluorescence**

| **Antibody** | | **Company (Cat. No.)** | **Working dilutions** | |
| --- | --- | --- | --- | --- |
| **TMEM117** | Proteintech Group (21314-1-AP) | | | WB: 1:500 |
| **ANP** | ThermoFisher Scientific (PA5-72527) | | | WB: 1:200 |
| **BNP** | ThermoFisher Scientific (PA5-79760) | | | WB: 1:200 |
| **β-MHC** | ThermoFisher Scientific (PA5-76225) | | | WB: 1:200 |
| **PERK** | Santa Cruz Biotechnology (sc-377400) | | | WB: 1:200 |
| **p-PERK** | Cell Signaling Technology (#3179) | | | WB: 1:1000 |
| **eIF2α** | Cell Signaling Technology (#9722) | | | WB: 1:1000 |
| **ATF4** | Cell Signaling Technology (#11815) | | | WB: 1:1000 |
| **GAPDH** | Abcam (ab181602) | | | WB: 1:5000 |
| **α-actinin** | Abcam (ab32575) | | | IF: 1:500 |

**Supplementary Figure Legends**

**Supplementary Figure 1** The knockout efficiency of TMEM117, blood pressure, hypertrophic marker genes and the ratios of HW/TL were determined. **(A)** Western blotting of TMEM117 expression in the heart samples from Control and TMEM117 cKO mice. **(B-C)** The systolic blood pressure (SBP) and diastolic blood pressure (DBP) in control mice and TMEM117 cKO mice after saline or Ang-II infusion for 4 weeks were determined by a noninvasive tail-cuff plethysmography. **(D)** The ratio of heart weight to tibia length (HW/TL) was determined in control mice and TMEM117 cKO mice after saline or Ang-II infusion for 4 weeks. **(E)** Transcript levels of hypertrophic marker genes (ANP, BNP, β-MHC) and fibrotic marker genes (Col1, Col3) were measured after saline or Ang-II infusion in control and TMEM117 cKO mice. **(F-G)** Western blotting was used to measure the transfection efficiency of si*TMEM117* in NMVMs (F) and Ang-II treated NMVMs (G). **(H)** The isolated neonatal mice ventricular myocytes NMVMs were authenticated by α-actinin staining (400X). cKO, conditional knockout; si*Control*, scrambled siRNA; si*TMEM117*, siRNA against mouse TMEM117. All the data represent the means ± SEM. N=6-8/group. ^*^*P*<0.05 and ^**^*P* <0.01. NS indicates no significance. Statistical significance was determined by 1-way ANOVA and 2-way ANOVA using Tukey post hoc test.


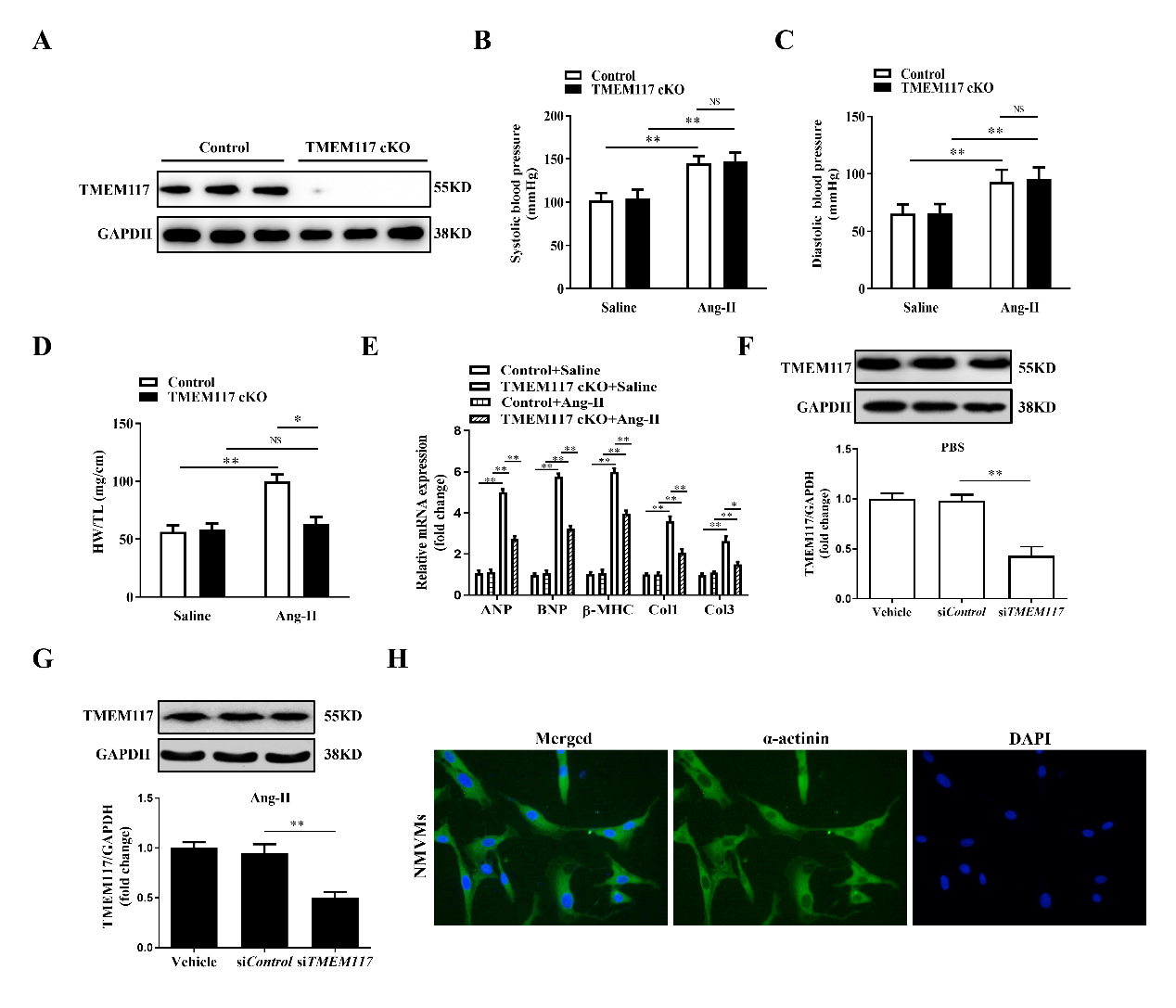


**Supplementary Figure 2** The overexpression of TMEM117, ratios of HW/TL and hypertrophic genes were measured. (**A-B**) Representative immunoblots and quantification of protein levels of MICU1 in Saline (A) and Ang-II groups (B) infected with Ad-EV and Ad-*TMEM117* were shown. (**C**) The ratios of HW/BW in control mice injected with Ad-EV or Ad-*TMEM117* subsequently subjected to saline or Ang-II were determined. (**D**) Transcript levels of hypertrophic markers and fibrotic marker genes in the indicated groups. (**E-F**) Representative immunoblots and quantification of protein levels of TMEM117 in (NMVMs) (E) and Ang-II treated NMVMs (F) infected with Ad-EV and Ad-*TMEM117* were shown. Ad-EV, control adenovirus; Ad-*TMEM117*, recombinant adenovirus encoding TMEM117. All the data represent the means ± SEM. N=6-8/group. ^*^*P*<0.05 and ^**^*P* <0.01. NS indicates no significance. Statistical significance was determined by 1-way ANOVA and 2-way ANOVA using Tukey post hoc test.


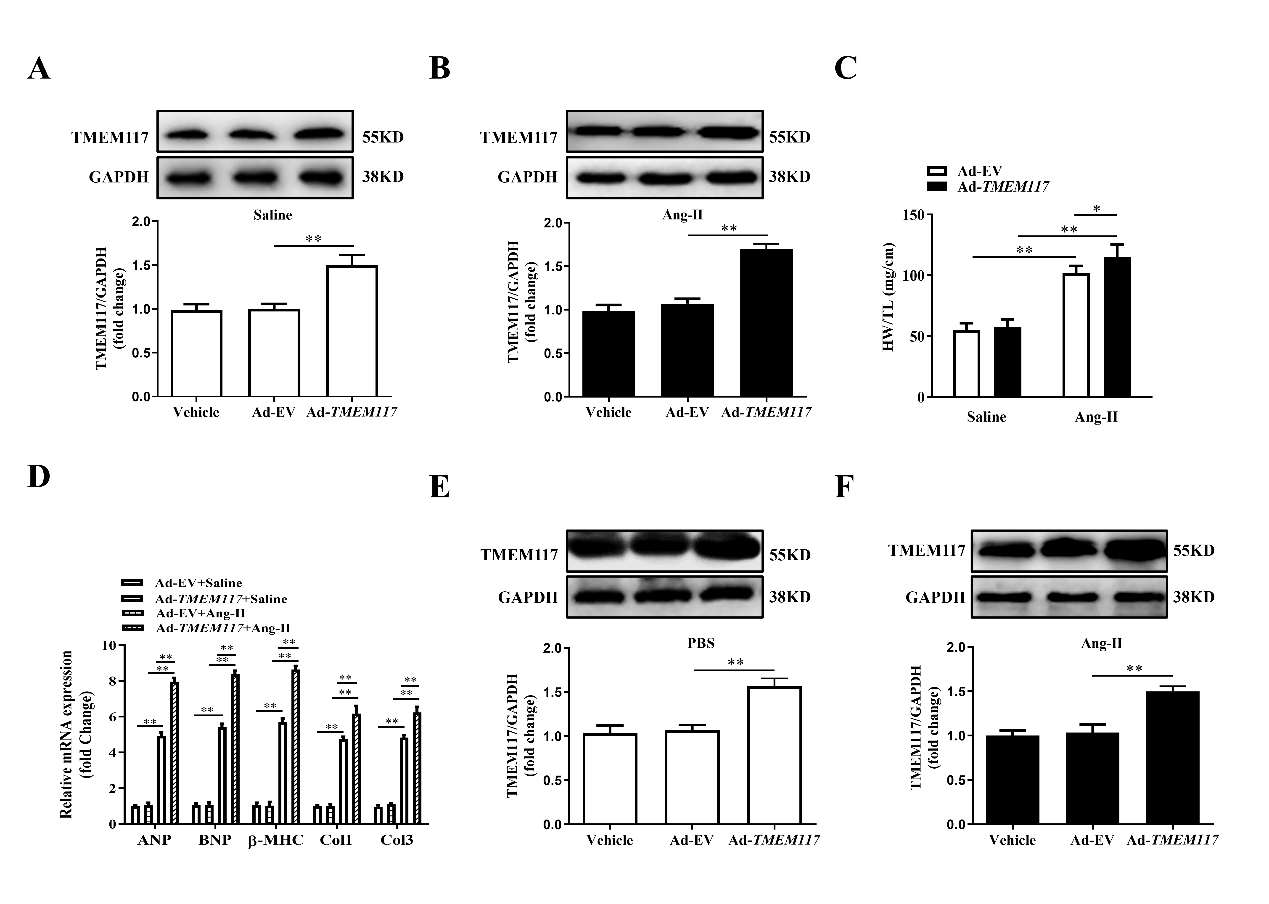

Supplement: Supplementary file 1 — Supplementary Materials [file 41440_2023_1377_MOESM1_ESM.docx]
